# Combining universal beauty and cultural context in a unifying model of visual aesthetic experience

**DOI:** 10.3389/fnhum.2015.00218

**Published:** 2015-04-28

**Authors:** Christoph Redies

**Affiliations:** Experimental Aesthetics Group, Institute of Anatomy I, University of Jena School of Medicine, Jena University HospitalJena, Germany

**Keywords:** experimental aesthetics, beauty, art, culture, painting, cognition, perception, emotion

## Abstract

In this work, I propose a model of visual aesthetic experience that combines formalist and contextual aspects of aesthetics. The model distinguishes between two modes of processing. First, perceptual processing is based on the intrinsic form of an artwork, which may or may not be beautiful. If it is beautiful, a beauty-responsive mechanism is activated in the brain. This bottom–up mechanism is universal amongst humans; it is widespread in the visual brain and responsive across visual modalities. Second, cognitive processing is based on contextual information, such as the depicted content, the intentions of the artist or the circumstances of the presentation of the artwork. Cognitive processing is partially top–down and varies between individuals according to their cultural experience. Processing in the two channels is parallel and largely independent. In the general case, an aesthetic experience is induced if processing in both channels is favorable, i.e., if there is resonance in the perceptual processing channel (“aesthetics of perception”), and successful mastering in the cognitive processing channel (“aesthetics of cognition”). I speculate that this combinatorial mechanism has evolved to mediate social bonding between members of a (cultural) group of people. Primary emotions can be elicited via both channels and modulate the degree of the aesthetic experience. Two special cases are discussed. First, in a subset of (post-)modern art, beauty no longer plays a prominent role. Second, in some forms of abstract art, beautiful form can be enjoyed with minimal cognitive processing. The model is applied to examples of Western art. Finally, implications of the model are discussed. In summary, the proposed model resolves the seeming contradiction between formalist perceptual approaches to aesthetic experience, which are based on the intrinsic beauty of artworks, and contextual approaches, which account for highly individual and culturally dependent aspects of aesthetics.

## Introduction

Research in the field of experimental aesthetics has gained much momentum in recent years. The roots of psychological research in aesthetics date back as far as to the 19th century (Fechner, 1876), but the research tools to tackle scientific questions in the field have become widely available to the scientific community only during the last decade or so. For example, modern brain imaging methods have empowered studies on how aesthetic experience correlates with the activation of brain regions in the human observer (for reviews, see Avram et al., 2013; Ishizu and Zeki, 2013; Vessel et al., 2013; Vartanian and Skov, 2014) and advanced computational methods permit the investigation of statistical image properties that are associated with aesthetically pleasing images (Datta et al., 2006; Redies et al., 2007b; Li and Chen, 2009; Graham and Redies, 2010; Braun et al., 2013).

One major problem in modern experimental aesthetics is that the subject of investigation, aesthetic experience, remains ill-defined. Artists and vision scientists alike have problems in narrowing down what aesthetic experience exactly is. Similarly, at the stimulus level, it remains unclear whether or not visual stimuli can have an intrinsic “significant form,” which can elicit the perception of beauty, as postulated by Bell (1914). If so, what is the nature of this beautiful form? Although many attempts have been made to define terms such as “artworks,” “aesthetics,” or “beauty,” their precise meaning is elusive. Moreover, for different types of visual stimuli, for example for images of human faces or bodies, landscapes and artworks, the same term may denote different things (Augustin et al., 2012). Despite the uncertainties, there seems to be a general agreement in the field that the scientific study of aesthetic experience and of beautiful stimuli holds great promise and may contribute significantly to our understanding of human brain function and behavior (Jacobsen, 2006; Chatterjee and Vartanian, 2014). In the present work, beauty refers to the inherent property of a visual stimulus; aesthetic refers to the subjective experience elicited by an artwork, or to the neural processing in the brain relating to that experience. Aesthetic experience consists of an intense feeling of pleasure, which people can have when viewing beautiful stimuli such as artworks. This general feeling should be distinguished from more domain-specific sensations of visual pleasure that are elicited, for example, by attractive faces, the beauty of natural scenes or fashionable clothing (see “Application of the Model to other Areas and Domains of Aesthetics”). Note that the term aesthetic experience has been used differently in some psychological theories to describe deeply moving and disrupting cognitive experiences that cause psychological and emotional adjustments in an individual’s lifetime; such experiences, which can evoke mental and emotional growth, do not necessarily involve sensual beauty (for example, see Lasher et al., 1983; Funch, 2007; Pelowski and Akiba, 2011).

In view of the many uncertainties in modern experimental aesthetics, models are crucial to develop testable research hypotheses. Scientific models have a great influence on the types of scientific studies asked. Current models in experimental aesthetics are inspired not only by scientific research, but also by ideas emanating from other fields, such as philosophical aesthetics, art history and art critique. Over the last two millennia, philosophers and art historians have reflected upon many of the fundamental questions that are advanced in experimental aesthetics today. They have done so with conceptual vigor and at great intellectual depth. On the one hand, this background has the great advantage that scientists can tap into a vast array of ideas about aesthetics. On the other hand, the proximity of experimental aesthetics to the other disciplines harbors the risk that experimental aesthetics emulates uncritically a narrow subset of philosophical positions. As always in science, it is important to keep an open mind in order to avoid being dogmatic about issues that have not yet been subject to rigorous scientific examination.

## Aim of the Proposed Model

At present, two major types of theories predominate in the scientific literature on experimental aesthetics (for a review, see Shimamura, 2014):

• *Formalist theories* propose that aesthetic experience relies on one or several formal properties of visual stimuli, in particular their intrinsic sensual beauty (Baumgarten, 1750; Bell, 1914; for a review, see Dowling, 2014). It has been argued that processing of beauty does not have to reach consciousness and may be largely non-verbal. Moreover, the formal properties are thought to be universal, i.e., they have the potential to elicit an aesthetic experience in viewers across human cultures and independent of the context of their creation (Bell, 1914). A more modern version proposes that the universal properties of artworks reflect basic mechanisms of human brain function (Zeki, 1999a), such as efficient coding principles (Redies, 2007).
• *Contextual theories* propose that aesthetic experience depends on the intention of the artist and the circumstances, under which the artwork was created and is displayed. Contextual theories focus on deliberate processing of explicit information that can be verbalized. Unlike image content, beauty plays only a minor role, if any. Contextual and content-based theories of art appreciation have been advanced in contemporary philosophical aesthetics in particular (Goodman, 1968; Dickie, 1974; Danto, 1981), as well as in empirical aesthetics (Jacobsen, 2006; Bullot and Reber, 2013; Zeki, 2013). Some of these theories focus on (post-)modern and contemporary art (Leder et al., 2004; Gopnik, 2012).

Two other types of theories play less of a role in current discussions in the field of experimental aesthetics and are not considered here in detail. First, mimetic theories stipulate that artworks mimic views onto reality and that the beholder evaluates the quality of artworks by how well it resembles real-world scenes. This idea cannot be applied to non-representational artworks. Second, expressionist theories emphasize that artists convey their feelings to the beholder through artworks and that it is the emotional quality of an artwork that is of paramount importance for aesthetic experience (for a review, see Shimamura, 2014). For a discussion of the role of emotions in aesthetic appreciation, see Section “Emotional Processing.”

Conceptual and formalist theories seem to contradict each other at first glance. On the one hand, some authors claim that content and context alone determine aesthetic experience and that, consequently, there is no intrinsic form in artworks that is preferred across cultures (Danto, 1981; Leder et al., 2004; Gopnik, 2012; Bullot and Reber, 2013; Zeki, 2013; Leder and Nadal, 2014). On the other hand, models that focus on the universality of beauty largely neglect contextual factors (e.g., Bell, 1914; Redies, 2007). However, as is common in science, aspects of both types of ideas seem germane to the question of what constitutes aesthetic experience, as pointed out by several researchers (see, e.g., Berlyne, 1971; Chatterjee, 2011; Van de Cruys and Wagemans, 2011; Sheridan and Gardner, 2012; Bullot and Reber, 2013; Graham, 2013; Mather, 2014). It remains unclear how the two opposing views can be combined in an integrative conceptual framework.

In the present work, I will present a unifying model of aesthetic experience, which integrates aspects of contextual and formalist theories. The model incorporates perceptual factors that determine the processing of beautiful form, as well as the cognitive processing of contextual variables that affect personal aesthetic preferences. It also accounts for aesthetic episodes elicited by (post-)modern art and by non-beautiful art. The model is dynamic and can explain changes in the reception of artworks over time. The model was primarily developed for the visual domain, but possible application to the other senses will be discussed also (see “Application of the Model to other Areas and Domains of Aesthetics”).

## The Model

**FIGURE 1:**
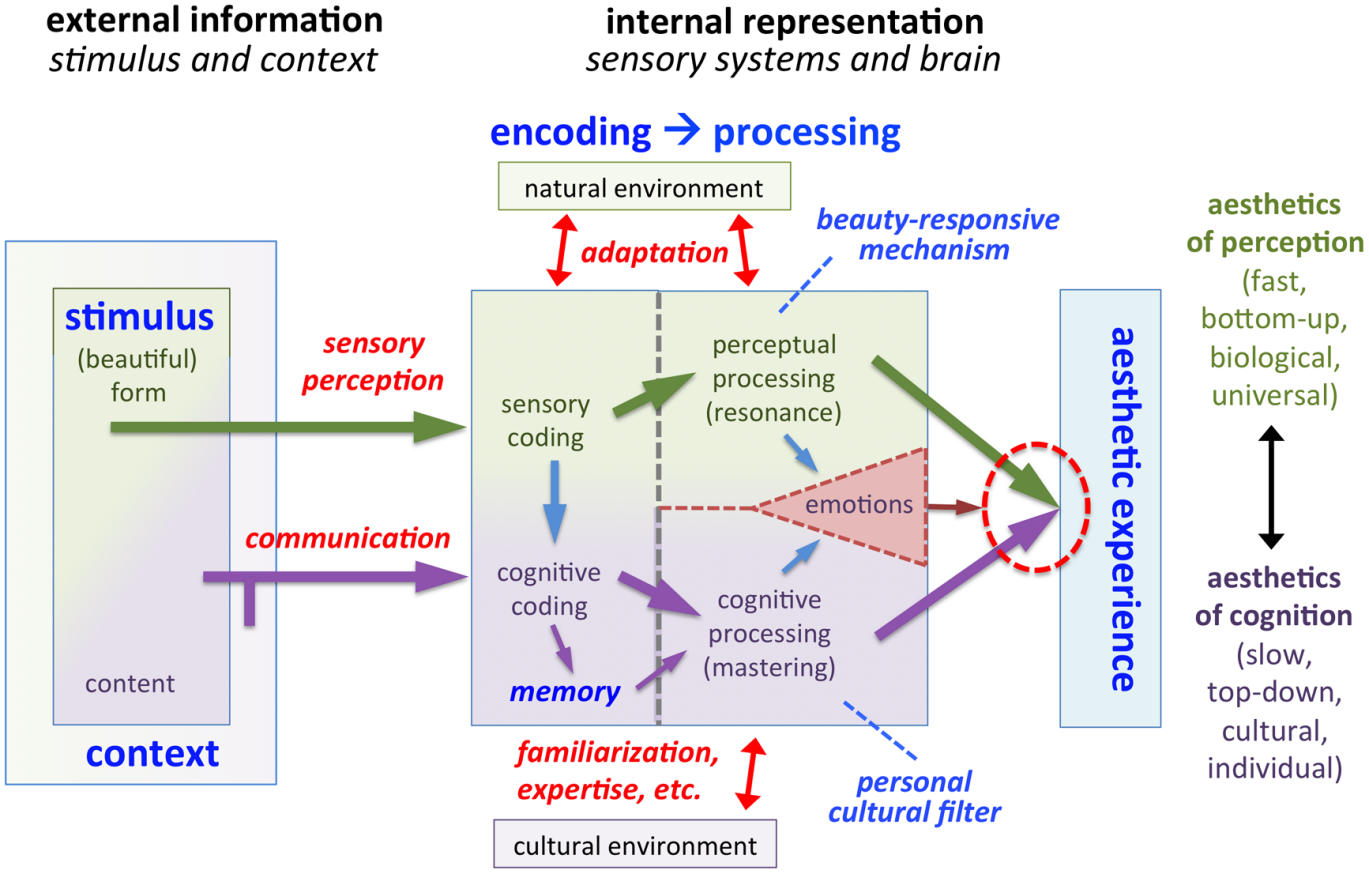
**Model of aesthetic experience**. The model distinguishes between *external information* (see “External Information”) and its *internal representation* in the nervous system of the art beholder or the artist (see “Internal Representation”). The external information consists of the *stimulus* (artwork) and the *context*, under which it was created or is viewed. From the left to the right, we proceed to the *encoding* of information in the nervous system. The arrows indicate the forward flow of information. Note that feedback loops and top-down processing, which take place in the cognitive processing stream in particular, are not indicated in the diagram. Stimulus *form* is encoded by mechanisms of *sensory perception* (see “Perceptual Coding and Processing”). The beholder gains cognitive information about the content and context of the artwork not only by viewing it but also by means of *communication*, for example, in a verbal dialog with another person or by reading a text (see “Cognitive Coding and Processing”). In addition, contextual information that has been obtained previously may be retrieved from memory. The initial encoding of stimulus form and context is then followed by further information *processing* in the brain. The thick arrows in the model represent two different channels of information processing: *perceptual processing* of form (green arrows at the top) and *cognitive processing* of content and context (purple arrows at the bottom). If processing in *both* channels is favorable (see “Joint Activation of Perceptual and Cognitive Channels”), i.e., if there is *resonance* in perceptual processing (*aesthetics of perception)*, and *mastering* of cognitive processing *(aesthetics of cognition*), aesthetic experience is induced (see “Aesthetic Experience”). *Emotions* can be elicited via both channels and modulate the degree of the aesthetic experience (see “Emotional Processing”).

**Figure 1** depicts my model of visual aesthetic experience. A brief outline of the model is provided in the legends to **Figure 1**. Following previous proposals (Sheridan and Gardner, 2012; Shimamura, 2014), the present model is based on the triad of cognition, perception and emotions (Chatterjee and Vartanian, 2014). Processing in the perceptual channel and cognitive channel takes place independently and in parallel; for aesthetic experience to be induced, successful processing in *both* channels is necessary (see “Joint Activation of Perceptual and Cognitive Channels,” which also provides examples of how the model works for different cases of aesthetic and non-aesthetic experiences). In Section “Predominant Activation of One Channel,” I will describe two special cases where processing in one of the two channels dominates the other. The third component of the model, emotions, can modulate the extent of the aesthetic experience (see “Emotional Processing”).

### External Information

The external factors of the model comprise the *form* and *content* of the stimulus as well as the *context* of its creation and presentation (**Figure 1**).

#### Stimulus

The stimulus in the model is a visual display of an artwork or an object, a visual scene or a visual pattern. In general, images of artworks are well demarcated from their visual surround. Often, a frame is used to enhance this boundary (Redies and Gross, 2013). Some visually pleasing stimuli, for example large vista views of beautiful landscapes, fill the entire visual field of the beholder’s eye. In any case, the stimulus of interest is usually viewed at the center of gaze, which falls onto the fovea of the retina, from where retinal ganglion cells project nerve fibers to the visual centers in the brain. About 80–90% of retinocortical connections and the subsequent processing in the visual cortex are devoted to the fovea so that peripheral vision contributes relatively little to processing in the brain. The stimulus may be static, or moving, such as in videos or dance. Information of stimulus movement is encoded already at the retinal level and is conveyed to the brain along a specialized channel (Wurtz and Kandel, 2000a).

##### Form

The *form* of the stimulus refers to *how* something is depicted in an image while image *content* refers to *what* is depicted. For example, an image may depict a bowl of fruit, which can assume different forms; it may be sketched by thin gray pencil lines, represented realistically in bright oil paint, outlined schematically in a coarse woodcut print, or fragmented in a cubist painting. Form includes both the local structure and the global (higher-order) structure of an image. Local structure corresponds to luminance or color and luminance contrast, lines edges, texture or shading cues that are present at a particular location of an image. In a digital image, it describes the relation of pixels within a narrow region of an image. Global structure refers to statistical regularities in large parts of the image or in the entire image, for example the spatial frequency content of the image, the kurtosis of its luminance values, overall complexity or self-similarity.

Form and content of an image are not independent of each other. For example, at the local level, an apple has a shiny, unblemished surface that is different from that of a hedgehog (many spikes). Also at the global level, images with different content may differ systematically in their higher-order statistical image properties. For example, images of most natural scenes possess a scale-invariant spatial frequency (Fourier) spectrum (Field, 1987; Tolhurst et al., 1992; Simoncelli and Olshausen, 2001) while images of printed text do not have such a scale-invariant spectrum (Melmer et al., 2013).

Before asking whether artworks have a characteristic intrinsic form or not, let us consider whether natural images and images of artworks represent a special subset of images in general. It has been argued that all natural images have a specific form and constitute a minute subset of all possible images (Ruderman, 1994; Simoncelli and Olshausen, 2001; Geisler, 2008; Graham and Field, 2009). For example, Graham and Field (2009) demonstrated that the vast majority of digital images looks like white noise, i.e., the gray values of each pixel appear more or less random and do not relate to those of neighboring pixels in the image. Humans cannot perceive any natural scenes or objects in white-noise images. In digital images of natural scenes or objects, the gray value of a given pixel is likely to be similar to that of the immediately adjacent pixels, i.e., neighboring pixels are highly correlated, because visual objects typically exhibit borders and surfaces that extent in a contiguous fashion across parts of the image. These statistical regularities in the pixel relations can be expressed in terms such as the pairwise correlation, kurtosis, collinearity, co-circularity etc. (Sigman et al., 2001; Simoncelli and Olshausen, 2001; Geisler, 2008; Graham and Field, 2009). The human visual system, in turn, is adapted in evolution and development to process these regularities with high efficiency and to extract biologically relevant information from them (see below).

Evidently, humans create artworks and other visual displays to be observed by humans. As a consequence, artworks are not perceptually indistinct like white noise images, but artworks resemble images of natural scenes or objects in that they have a form or structure that can effectively stimulate the human visual system (Redies, 2007; Graham and Redies, 2010). In conclusion, like natural images, most artworks likely tend toward a form suited for human vision.

However, not all images created by humans for humans are beautiful, just because they can stimulate some perceptual mechanism efficiently. For example, a bright red dot, yellow dot and green dot placed in a vertical line and presented in isolation on a dark background are excellent stimuli for our vision system, but most people would concur that they look like a traffic light that is not highly beautiful. Another example: In their paintings, impressionist artists have focused on the stimulation of color and shading mechanisms in vision, but not every painting produced in the impressionist style is highly beautiful. More complex visual effects have also been used in some specific types of art. For example, in the peak shift effect, animals respond more strongly to exaggerated stimuli than to normal ones. This principle has also been cited for the sexual features in some types of Indian sculptures (Ramachandran and Hirstein, 1999). Lastly, regular printed text is a stimulus that has been optimized for reading but it is not necessarily beautiful (Melmer et al., 2013).

In summary, the natural environment projects images with particular statistical regularities onto the retina. These image regularities are efficiently processed by specialized neural mechanisms in the visual system. We can thus expect that visual artworks have specific image properties because they were created so that their form can activate perceptual mechanisms in the human visual system (Redies, 2007; Graham and Field, 2008; Graham and Redies, 2010). Therefore, *both* natural images and artworks represent a subset of all images. The above conclusion does not help us, however, to decide whether beautiful images possess a specific form and represent a subset of all natural or man-made images. This question will be addressed in Section “What is the Nature of the Beauty-Responsive Mechanism and Which Stimulus Features Activate It?”

##### Content and context

The *content* of an image refers to anything that can be recognized by the viewer as persons, other natural or inanimate objects, and their actions or relations to each other. Content may also refer to emotional, social, political or philosophical concepts that artists intend to convey though their artworks. In general, image content can be made explicit, i.e., it enters consciousness as a meaningful interpretation of what we see, and it can be described in everyday (non-technical) language in most cases.

I have no doubt whatsoever that cultural factors and the previous experience of the artists and the art viewer have a crucial effect on the depiction and interpretation of content in artworks. In order to understand the meaning of artworks and the intentions of the artist, the art viewer must have some understanding of the cultural and individual circumstances, under which the artwork was created. As a consequence, the evaluation of image content depends on the *context* of the artwork’s creation. Likewise, the context of the presentation of an artwork (e.g., museum, laboratory, or book) has an effect on the aesthetic experience of the beholder (see, for example Gerger et al., 2014). Both content and context are factors that are shaped by the cultural environment and the individual experience of the artist and the art viewer. Content-based and contextual features that affect aesthetic experience include familiarity, prototypicality, novelty, originality, expertise, satisfaction, ambiguity, personal taste, interest, arousal, art-historical information, artistic understanding, social status and financial interests. A detailed account on how these diverse explicit features can affect the creation of artworks and the aesthetic experience has been published, for example, by Leder et al. (2004; modified in Leder and Nadal, 2014) and by Jacobsen (2006) for diverse psychological factors, and by Bullot and Reber (2013) for the art-historical context.

Artistic style is another feature of artworks that is strongly affected by cultural context. Style is important not only for how things are depicted in artworks but also for the subject matter depicted. For example, 16th century medieval paintings make use of a particular type of perspective, a limited set of colors and usually depict religious subject matters. This style is different from, for example, expressionist paintings that are characterized by wide variety of subject matters and emphasize the subjective emotional experience of the artist. In general, stylistic matters are subject to conscious decisions by the artist and depend largely on cultural context.

Last but not least, different techniques can be used to create artworks (oil painting, watercolor, etching, woodcut, etc.). Artistic styles have a large influence on which techniques artists use, but artists may depict the same content by using different techniques. For example, Edvard Munch created several versions of “The Scream” (oil painting, lithograph, and pastel drawing). A particular technique may tend to come along with specific local image properties, but these image properties do not by themselves determine whether an image is beautiful because, in principle, any technique can be used to create artworks.

### Internal Representation

In the model, *sensory coding* and *cognitive coding* are defined as the translation of external information into neural activity and they are a prerequisite for further information processing in the brain. The form of external stimuli is encoded by mechanisms of *sensory perception* that take place in the retina and represent, at the same time, the lowest stages of visual processing. The other two features of external stimuli in the model, content, and context, are encoded in the nervous system by *cognitive coding*, but not necessarily through the visual channel alone (see above). Once information is internally represented in the nervous system, it is *processed* to extract and enhance relevant information. Contextual information in particular is also stored in *memory* to be retrieved later.

#### Perceptual Coding and Processing

##### Perceptual coding of form

From the retina to the primary visual cortex, specific local features of visual images, for example, luminance contrast, color contrast, edges, three-dimensional depth cues and movement in visual scenes are detected and enhanced. To a large degree, the underlying visual mechanisms are driven bottom–up, take place automatically at lower stages of visual processing and are fast. Sensory coding and basic processing are mediated by neuronal mechanisms that are universal amongst humans; during the lifetime of an individual, these mechanisms can be modified by visual experience to a limited degree under normal circumstances, e.g., by adaptation (Wurtz and Kandel, 2000c).

Not all external signals can be encoded by the sensory systems. For example, humans cannot perceive ultraviolet or infrared light at the extremes of the spectrum of visible light. Consequently, it would make little sense for an artist to paint an artwork with ultraviolet color because nobody would be able to see it under natural lighting conditions. By the same token, a completely color-blind person will not be able to appreciate fully the artworks of French impressionists or other colorful artworks. Moreover, vision is an active process, by which the human visual system extracts visual information based on attentional mechanisms. We can assume that the perceptual mechanisms that enable us to orient ourselves in our daily life represent an *adaptation* to the visual structure of our natural environment during evolution (for a review, see Simoncelli, 2003) and during an individual’s lifetime. For example, movement is an important visual feature and the human visual system possesses several mechanisms to encode and process this particular aspect of visual information with high efficiency and accuracy. Similarly, the preference for particular colors helped our ancestors to survive better, probably because color vision proved useful in their search for food, such as ripe fruit (for a review, see Palmer et al., 2013). The visual system is thus composed of mechanisms that process visual information in a particular fashion. Such mechanisms include, for example, contrast enhancement of luminance and color, adaptation, movement-sensitive neural circuits, depth perception etc. The modification of visual information by the visual system can be studied in numerous visual illusions that enable us to experience directly the difference between physical stimuli and our perception of them.

In general, artists have a good intuitive or explicit knowledge of how we perceive and how the brain processes visual information. This knowledge allows them to create artworks that can efficiently stimulate the retina and visual brain (Cavanagh, 2005). Artists used some visual effects in their works long before vision scientists discovered them in their research. For at least a century, there has been a very lively exchange between artists and vision scientists on how we perceive the external world. This mutual exchange has been beneficial to both sides (Spillmann, 2007) and there are several scientific publications that describe this exchange with abundant illustrations (Gregory et al., 1995; Zeki, 1999b; Livingstone, 2002).

**FIGURE 2:**
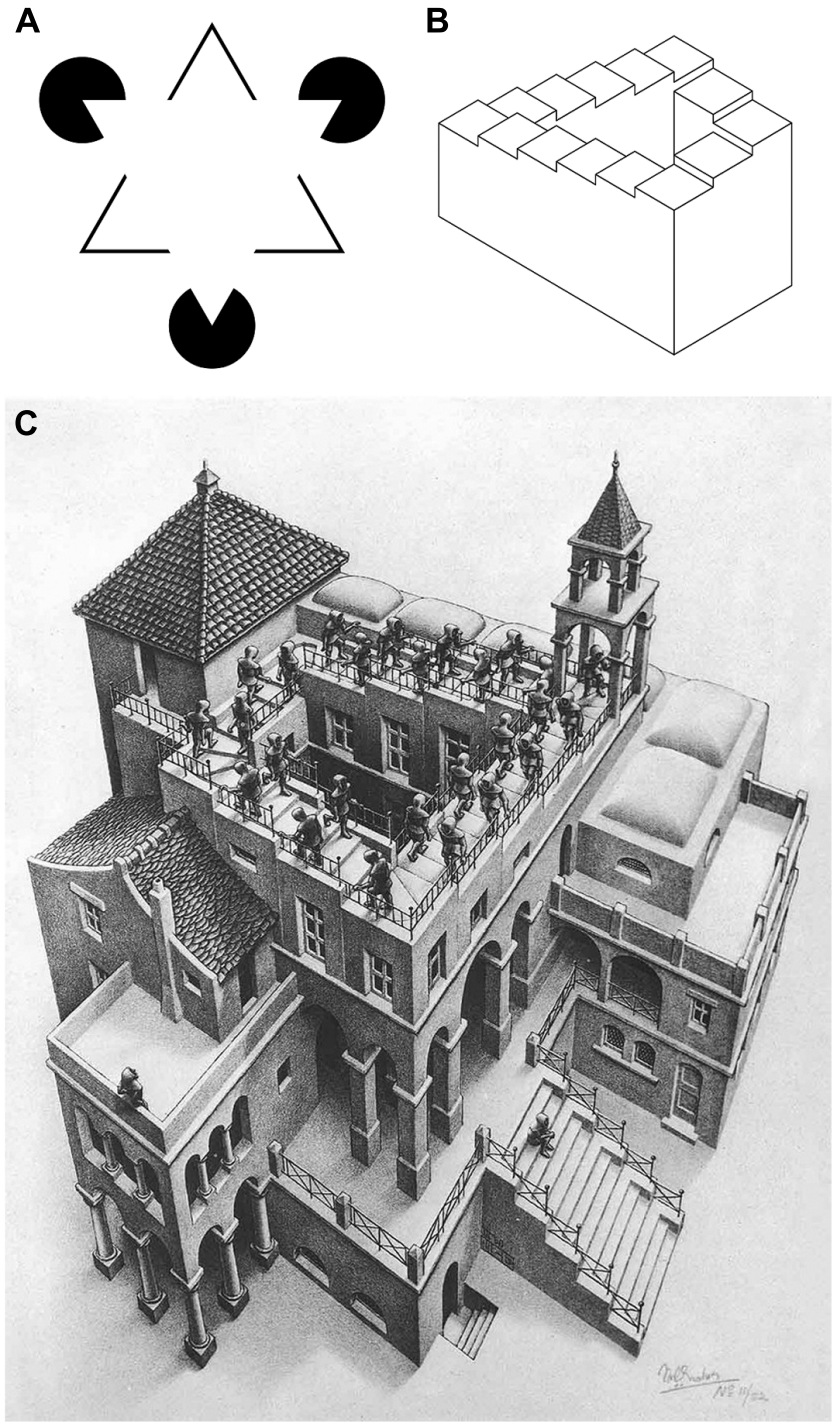
**Examples of visual effects (illusions). (A)** Kanizsa’s (1955) triangle. Note the bright and sharply demarcated illusory triangle that is induced by spatially separate black circles (pac-mans) and the segmented outlines of a triangle. **(B)** Impossible staircase (Penrose and Penrose, 1958). This object is impossible in three-dimensional space because the steps continuously descend or ascend. **(C)** A similar visual effect was used by M. C. Escher in his artwork *Klimmen en Dalen* (1960). Note that the image showing the isolated effect is not a beautiful artwork while Escher’s lithograph is **(C)**. M. C. Escher’s “*Klimmen en Dalen*” © 2015 The M.C. Escher Company – The Netherlands (www.mcescher.com). All rights reserved. Used by permission.

However, the illustration of a particular visual phenomenon alone is not sufficient to create a visual artwork. Straightforward displays of visual illusions, such as the Kanizsa (1955) triangle (**Figure 2A**), can hardly be considered works of art. If artists use such visual elements in their works, they usually do so in an intricate and complex way that surpasses the simple illustration of the visual phenomenon. For example, the Dutch artist M. C. Escher used Penrose’s impossible staircase (**Figure 2B**; Penrose and Penrose, 1958) in his 1960 lithograph “*Klimmen en Dalen*” (**Figure 2C**). This lithograph is a work of art not only because it illustrates a particular visual effect, but also because of the overall artistic composition of the artwork. Note that the impossible staircase constitutes only a small part of the artwork; its illusory nature is noticed after closer inspection of the people climbing and descending the stairs and may provide an example of a cognitive ‘aha’ effect that artworks can elicit by cognitive mastering (Lasher et al., 1983; Pelowski and Akiba, 2011; Muth and Carbon, 2013). The perceptual quality of this lithograph, however, stems from the gestalt of the entire composition, not just from the depiction of the staircase.

Vice versa, if scientists study special visual effects that artists use in their paintings, this endeavor should not be confused with experimental aesthetics. Research in experimental aesthetics aims to study the basis of aesthetic experience and its neural correlates, whereas elementary mechanisms of visual perception are the subject of vision science in general. Studying general mechanisms of visual perception may be useful and interesting to both scientists and artists, but it does not help us to understand the difference between ordinary visual stimuli and artworks.

##### Higher-level perceptual processing

It is well established that, at low levels of the visual system (up to the primary visual cortex), information processing is predominantly local, i.e., it is restricted to small regions of the retina (Wurtz and Kandel, 2000b). Local visual information, such as luminance, color and movement, are passed along to the visual cortex in separate channels. In the primary visual cortex, this information is then mapped retinotopically onto the so-called hypercolumns, which process local information from small regions of the visual field. In higher visual cortical areas beyond the primary visual cortex, information is merged more and more from larger regions of the visual field to extract more global features. At the same time, the higher visual cortical areas become specialized in processing specific aspects of visual information, for example, color in cortical area V4, movement in the medial temporal cortical region, and faces in the fusiform gyrus (Wurtz and Kandel, 2000b).

Assuming that correlates of the perception of beautiful form exist, these findings bring up two questions: (1) Are the correlates more likely to be found at lower or at higher levels of the visual system? (2) In which of the different visual channels or cortical areas can we anticipate to encounter the correlates? The answer to both questions is relatively straightforward.

First, there is strong evidence that the perception of beautiful form depends, at least in part, on global image properties. The commonly used term “composition” clearly refers to the relation of visual elements (lines, edges, surfaces, etc.) across an artwork. In a beautiful artwork, local elements are integrated into an overall gestalt that pervades the entire artwork and puts each pictorial element in perceptual relation to the other elements in the artwork. As a consequence, the global integrity of an artwork is paramount for the appreciation of its beauty. If beauty were a purely local phenomenon, it would be possible to cut an artwork into pieces and enjoy the pieces one by one on different days. Reassembling the pieces of an artwork randomly has as much a deleterious effect as has punching a sizable hole into a painting. Even more detrimental would be to exchange parts between different paintings. Of course, such manipulations may have an effect not only on the form but also on the content of an image, but one could think of options (e.g., for abstract art) that selectively affect image form without changing image content (McManus et al., 1993; Redies et al., 2015).

Second, it is even more evident that the perception of beauty is not restricted to one particular mechanism of visual processing. Rather, in the history of art, it has been shown over an over again that beautiful artworks can be created in almost any style and by using any variety and combination of visual effects, for example, color, luminance edges, depth cues, occlusion, movement, adaptation, visual ambiguity, and so on. Related to this variety of visual mechanisms, the material that can be used to create beautiful objects seems limited only by practical constraints but not by artistic ones. Consequently, neural mechanisms that detect beauty in visual stimuli must be localized in most if not all cortical regions that process visual information. While it is theoretically possible that, for each of the different visual regions or mechanisms, there is a different type of neural correlate of beauty perception, it seems more economical to propose a single and common neural mechanism, which I call the *beauty-responsive mechanism* in my model.

**FIGURE 3:**
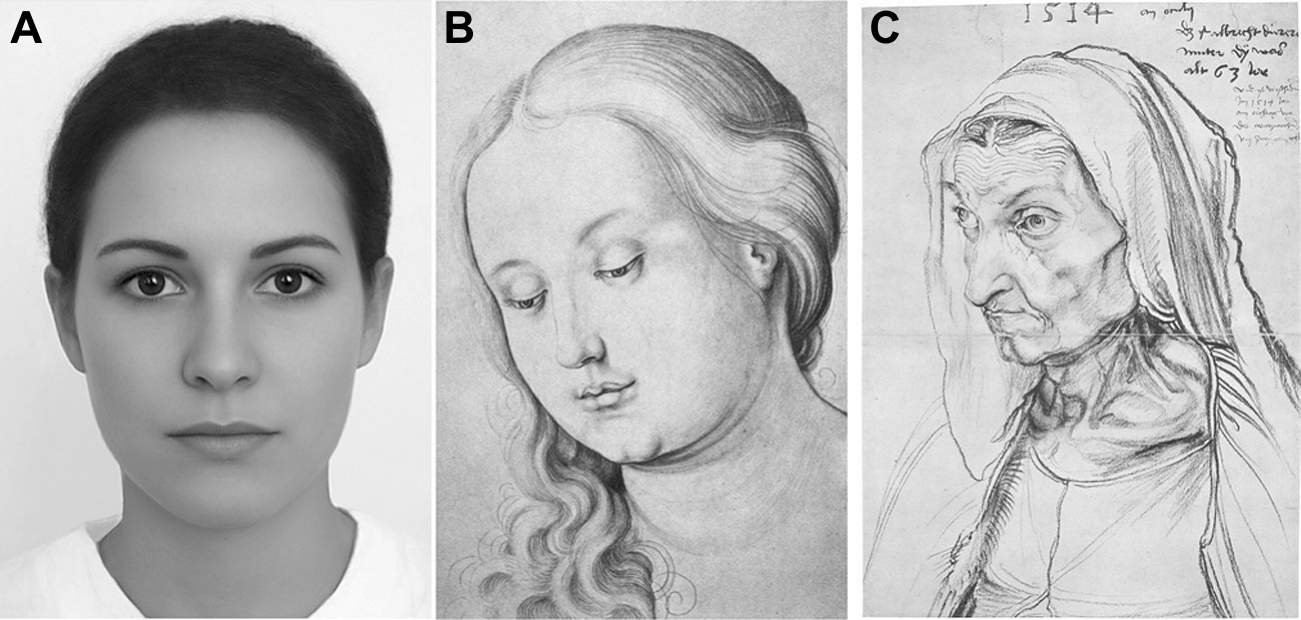
**(A)** Average faces generated by morphing and superimposing veridical images of female faces. **(B)** *Portrait of a Young Women* by H. Baldung Grien, about 1513–1515. **(C)** *Portrait of the Artists’s Mother at the Age of 63* by A. Dürer, 1514. The figure illustrates the difference between the (domain-specific) face attractiveness (in **A,B**) and the (general) intrinsic beauty of artworks (in **B,C**). Note that the drawing in **(C)** is beautiful, despite the fact that the depicted face is not attractive. The image in **(A)** was reproduced with kind permission by © Martin Gründl (www.beautycheck.de, accessed January 28, 2015).

By postulating a general beauty-responsive mechanism, I do not deny that there are general preferences for particular types of stimulus properties in the different visual domains. For example, in the color domain, people in Western cultures generally prefer bluish and greenish colors and dislike brownish ones (Yanulevskaya et al., 2012; Mallon et al., 2014), possibly because blue and green can be associated with pleasant scenes (lakes, sky, forest, flowers) while disgusting things like excrements or rotten food have a brownish color (Palmer and Schloss, 2010). Despite this general color preference, an etching printed in a brownish color can nevertheless be a beautiful piece of art. As another example of domain-specific beauty, people across cultures prefer particular features in female and male faces (symmetry, smooth skin, particular facial proportions etc.; Rhodes, 2006). Not all images of attractive faces, however, are necessarily great artworks and display a beautiful artistic composition. **Figure 3** shows an image of an attractive average face that most observers would not classify as an artwork (**Figure 3A**), a somewhat attractive face in a beautiful artwork (**Figure 3B**) and an ugly face in a beautiful artwork (**Figure 3C**). These images demonstrate that facial attractiveness and the formal beauty of an artwork are not the same (see also Hayn-Leichsenring et al., 2013). Typically, domain-specific beauty, such as facial attractiveness, depends on what it depicted in an image while the formal beauty of artworks does not.

##### What is the nature of the beauty-responsive mechanism and which stimulus features activate it?

If the same beauty-responsive mechanism operates in different cortical areas, it seems reasonable to assume that this mechanism relates to some basic and general principles of neural processing. Basic mechanisms of information processing are, for example, long-range spatial inhibition, short-term adaptation, facilitation, long-term potentiation and depression, predictive coding or efficient (sparse) coding of information. Although it is at present unclear which – if any – of these mechanisms correlate with the perception of beauty, preliminary observations point in the following directions.

First, evidence is accumulating that large subsets of artworks of Western and Far Eastern provenance share a scale-invariant Fourier spectrum that has a spatial frequency profile similar to those of complex natural scenes (Graham and Field, 2007; Redies et al., 2007b; Alvarez-Ramirez et al., 2008). Accordingly, Western artists tend to portrait faces with the scale-invariant spectrum of natural scenes although photographs of faces have a different spectrum with a lower proportion of higher spatial frequencies than natural scenes (Redies et al., 2007a). Scale invariance indicates that the Fourier spectrum does not change as one zooms in and out of an image. The similarity between details of an image and the entire image has also been assessed more directly by comparing histograms of oriented luminance gradients at different levels of image detail (Amirshahi et al., 2012). With this approach, a relatively high degree of self-similarity was found for images of artworks and images of natural patterns (Redies et al., 2012). Particularly striking is the fact that the American abstract expressionist, Jackson Pollock, painted images with a fractal (self-similar) structure (Taylor, 2002; Taylor et al., 2011) even before Mandelbrot (1975) discovered the concept of fractality. Although J. Pollock was aware of the general visual resemblance of his paintings with natural patterns, he did not characterize this similarity in any explicit detail.

The similarity between artworks and natural patterns is of interest also because, like artworks, natural scenes can be beautiful, although perhaps not as profoundly as artworks. Several neurophysiological and psychological studies indicate that the mammalian visual system is adapted in evolution and ontogenetic development to process complex natural stimuli with a relatively high degree of efficiency compared to other types of stimuli (Olshausen and Field, 1996; Párraga et al., 2000; Vinje and Gallant, 2000; Simoncelli and Olshausen, 2001). It has therefore been speculated that beautiful artworks are created so that they can be processed in a way that relates to the coding of natural stimuli (Redies, 2007; Graham and Redies, 2010). Such a perceptual resonance between artworks and the visual system might elicit a specific pattern or state of neural activity in the visual system when an individual is observing a beautiful artwork. At present, however, it remains unclear what this particular state or resonant pattern of neural activity might be.

Second, complexity is another visual feature that has been linked to aesthetic preference. Although there are many ways to define visual complexity, most studies agree that an intermediate degree of complexity is preferred for artworks on average (Berlyne, 1974; Forsythe et al., 2011; Redies et al., 2012). For example, Taylor and colleagues systematically modified the fractal dimension, a measure that reflects visual complexity, in natural and synthetic visual patterns. They showed that patterns with a fractal dimension of about 1.3–1.5 are generally preferred over patterns with a higher or lower fractal dimension (for a review, see Taylor et al., 2011). However, the range of complexity values of artworks is relatively large (Braun et al., 2013).

A third and unexpected finding is that some of the statistical regularities investigated, specifically the spatial frequency spectrum and the oriented gradients in an image, are relatively evenly distributed across the orientations in artworks, i.e., they are isotropic (Koch et al., 2010; Braun et al., 2013; Melmer et al., 2013). The significance of this finding remains unclear. Some natural scenes and natural patterns possess a similarly high degree of isotropy.

Last but not least, a fourth formal feature of artworks is pictorial balance with respect to the center of an image, although this feature is more difficult to define in physical terms. Arnheim (1982) pointed out the importance of the image center for artistic composition. McManus et al. (2011) described evidence that the center-of-mass of luminance values was close to geometrical lines (horizontal, vertical, and oblique) in high-quality photographs. When Locher et al. (2005) studied the contribution of color to the subjective center of balance in original and modified Mondrians, they found the center closer to the geometrical center in original Mondrians than in modified ones.

I must point out, however, that none of the visual regularities of artworks mentioned above are necessary or sufficient to induce an aesthetic experience. For example, highly self-similar or fractal images can be produced by computer programs (Lee and Mumford, 1999) or by shuﬄing the phase of Fourier-transformed fractal images. The resulting images look vaguely natural, but they are not very beautiful. Likewise, an intermediate degree of complexity does not guarantee that a stimulus is beautiful. The “significant form” of beautiful artworks postulated by Bell (1914) therefore remains enigmatic up to date.

In summary, some of the evidence available to date suggests that the visual structure of beautiful artworks possibly reflects an adaptation of the human visual system to the natural environment. In Section “Form,” I have argued that images of natural stimuli and beautiful artworks represent a subset of all possible images, which can be readily encoded and processed by the human visual system. Here, I go beyond this claim and propose that beautiful images are a subgroup of all man-made images. Beautiful images are special because they possess a specific form that harbors the potential to induce a resonant aesthetic state of neural activation in the beholder’s visual system. This state reflects basic principles of neural processing and can be induced in various brain regions. Hence, visual art may embrace all modalities of visual perception.

Opponents of the notion that beautiful artworks have a specific form will only be convinced of its existence if this form is unequivocally demonstrated. Understandably, many researchers remain doubtful because attempts to describe this form in concrete terms have failed so far. However, this failure may be due to the fact that basic mechanisms of neural processing, such as efficient processing or sparse coding, are not readily accessible to cognitive introspection. In the same vein, the compositional rules for creating beautiful artworks also seem to be beyond the reach of cognition or everyday language. For this reason, it has been impossible to precisely define what renders an image beautiful in common language. Rather, we will rely on studies of formal and abstract physical image properties as well as neurophysiological recordings from the visual system to investigate in the future which image properties activate the putative beauty-responsive mechanism.

#### Cognitive Coding and Processing

Not only the perceptual abilities, but also the cognitive facilities of the human brain are limited. Like every other organ, the human brain is the result of an adaptation to the natural environment of our ancestors. Therefore, in the Darwinian sense, the neural substrates of cognition served specific functions in the survival of the human species, even if the original suitability of some of the cognitive functions is no longer apparent in our modern world. As pointed out by Dissanayake (2008), culture may have a biological basis also and humans are born with a preparedness to become cultural. This inborn preparedness manifests itself, for example, in language acquisition or face learning in early infancy (Tomasello, 1999). Nevertheless, for the purpose of the present discussion, it seems feasible to distinguish between biological (perceptual) mechanisms and cultural determinants of aesthetic experience.

The cultural environment determines our language, social behavior, aesthetic preferences and so forth. Cultural practices change slowly over the years and vary across cultures. Cognitive processing is shaped also by the experience gained during the lifetime of an individual. In my model, I distinguish between cognitive coding and cognitive processing. With regard to the art viewer, cognitive coding refers to the acquisition of explicit information about what is depicted in an artwork, the circumstances under which the artwork was created, the way it is presented and so on. Knowledge relevant for the cognitive evaluation of an artwork may also be stored in and retrieved from memory, based on previous exposure to art (**Figure 1**). Cognitive processing refers to the conscious usage of this information in order to understand the meaning of the artwork and the intentions of an artist. The artist, in turn, purposely uses visual signs or symbols that convey explicit content and meaning in his artworks.

As already mentioned in Section “Content and Context,” the cultural context and the previous personal experience of the artists are of paramount importance for the creation of artworks, especially for their content. Cultural and personal factors play an important role also when humans observe artworks. Because both the cultural environment and the interaction of an individual with this environment are highly variable within a society, the cultural experience of artists and art viewers alike is always intertwined with the special individual viewpoint of a person.

There are abundant descriptions and models on how contextual factors determine the creation of artworks and the aesthetic experience when observing artworks. The issue is also central to art history and the literature in this field is far too vast to be reviewed here. Two exemplary contextual theories in the field of empirical aesthetics are the multistage psychological model by Leder et al. (2004; revised in Leder and Nadal, 2014) and the psycho-historical framework by Bullot and Reber (2013).

In the present model (**Figure 1**), the different contextual factors that contribute to the evaluation of the content of artworks are incorporated in the form of the *personal cultural filter* (Redies, 2007). If the cognitive and cultural settings of the filter are compatible with the contextual information that is provided for a given artwork, the filter is permissive and allows for the successful processing of the contextual information, eventually leading to the cognitive mastering of the artwork’s content and context. If the setup of the cultural filter does not contain the cultural expertise to understand the artist’s intentions, cognitive mastering fails. Such a failure may induce a re-evaluation process that leads to self-evaluation and self-change, personal growth and eventual return to cognitive mastering with a new set of expectations (Lasher et al., 1983; Pelowski and Akiba, 2011).

### Joint Activation of Perceptual and Cognitive Channels

In the above sections, I described how the external information associated with an artwork is encoded by the nervous system and processed in the brain along two separate streams, which encode and process the perceptual and cognitive attributes of the artwork. In this section, I will explain how the two streams interact. As a central mechanism of the model, I propose that, in order to trigger an aesthetic experience, both channels must fulfill a specific condition (red dotted circle in **Figure 1**). The model thereby combines elements of formalist theories and contextual theories of art. The necessary conditions are:

• Perceptual processing: the neural mechanism responsive to beauty is activated in the visual brain region(s), in which the processing of the visual stimulus takes place (*perceptual resonance*; see “What is the Nature of the Beauty-Responsive Mechanism and Which Stimulus Features Activate It?”). It is activated directly by intrinsic formal properties of artworks. Processing is predominantly automatic, fast and proceeds in a bottom–up direction.
• Cognitive processing: the personal cultural filter is permissive, i.e., the content and context of the artwork are successfully and positively evaluated, which results in *cognitive mastering* (see “Cognitive Coding and Processing”). The personal cultural filter is a mental construct that is shaped by the experience of an individual in a cultural context. Cognitive processing is generally slower than perceptual processing. Top–down mechanisms, which mediate expectations or involve the retrieval of contextual information from memory, play an important role; note that they are not explicitly indicated in **Figure 1**.

**FIGURE 4:**
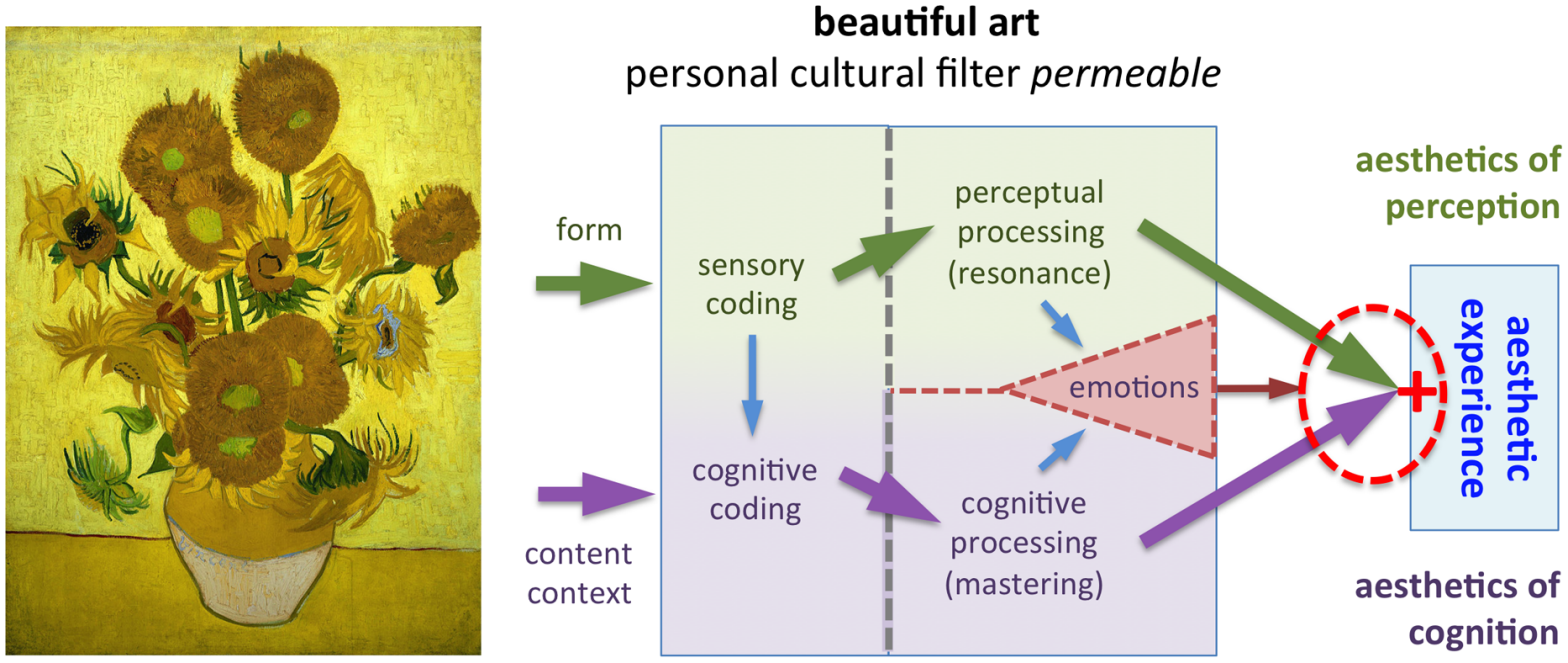
**Example of a beautiful artwork that can elicit an aesthetic experience (*Sunflowers* by van Gogh, 1888)**. The beautiful form of this painting activates the beauty-responsive mechanisms in the perceptual processing stream (green arrows). Because van Gogh’s art style and life are well-known to most persons who view this painting, mastering is possible in the cognitive processing stream (purple arrows) so that the personal cultural filter is permeable. Hence, both conditions for an aesthetic experience are fulfilled.

**FIGURE 5:**
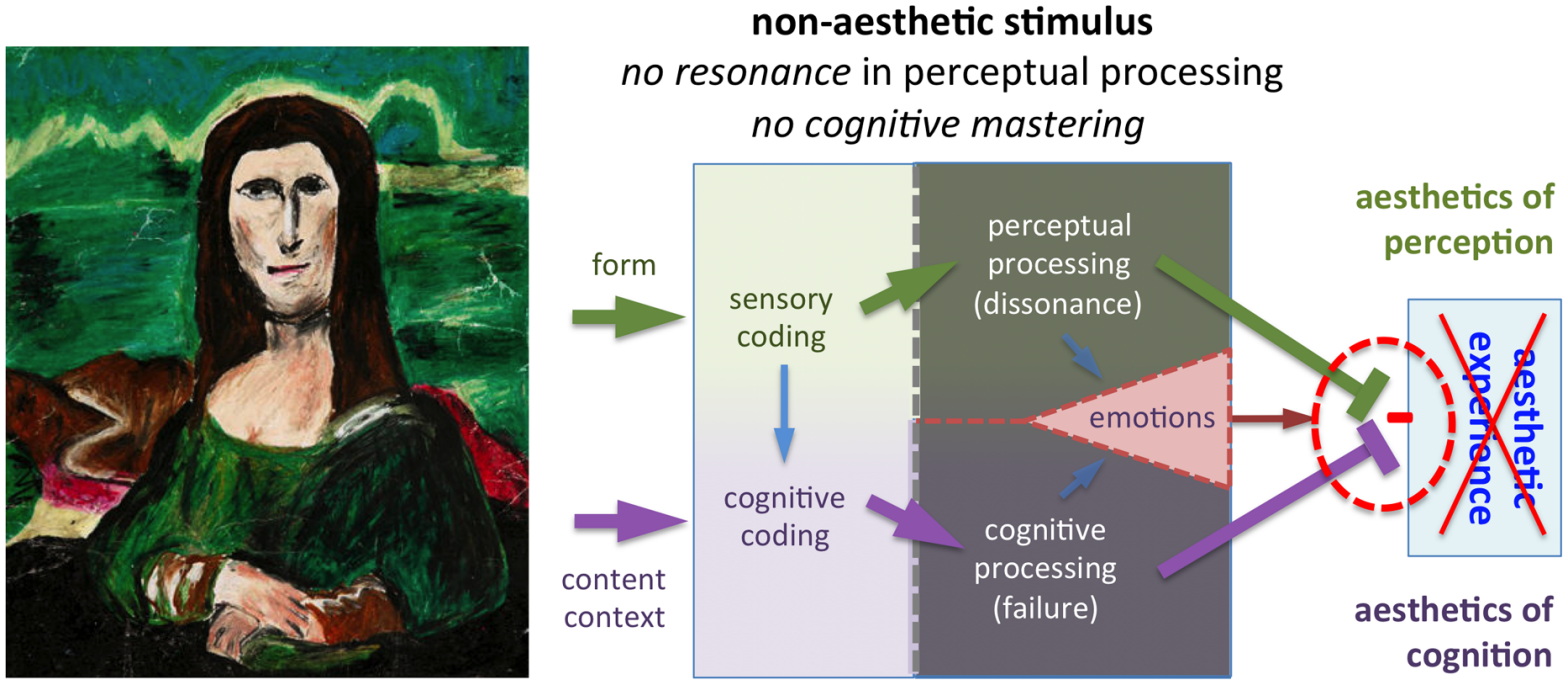
**This painting (*Mana Lisa* by A. Schmidt) is a copy of the Mona Lisa painting by Leonardo da Vinci (1503–1506)**. Because it is readily recognized as a copy produced by an unskilled artist and the intentions of the artist are not clear, there is no cognitive mastering for most individuals. Intrinsic beauty is very limited and resonance in the perceptual processing channel is at a low level, if not absent. The gray shading indicates unsuccessful perceptual and cognitive processing. As a result, there is no aesthetic experience. Reproduced with kind permission by © The Museum of Bad Art (MOBA), 2014.

**FIGURE 6:**
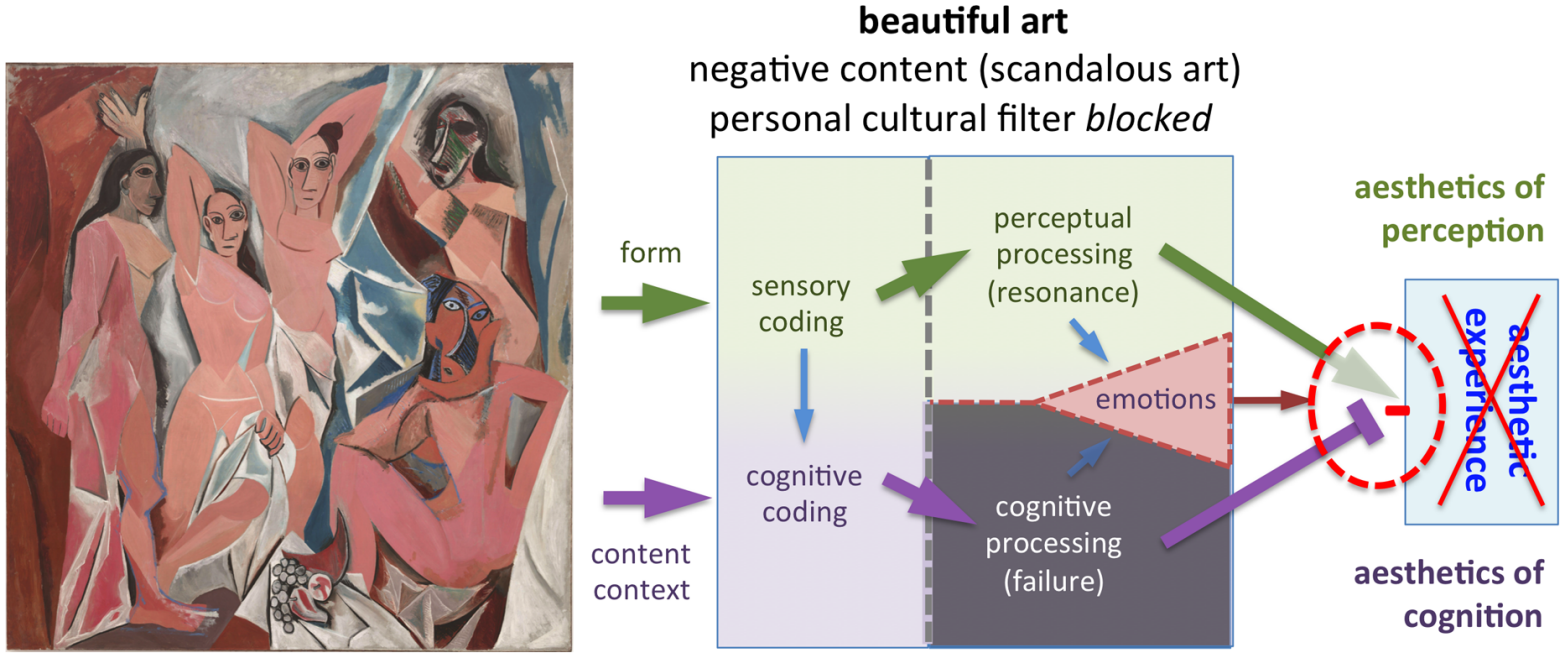
**Example of scandalous art (*Les Desmoiselles d’Avignon* by Picasso, 1907, © Succession Picasso/VG Bild-Kunst and Bonn, 2015)**. The brothel scene depicts five prostitutes. The painting is an early cubist painting and stands for a radical departure from conventional European painting style. The gray shading indicates unsuccessful cognitive processing. At the time when the painting was first presented, it caused a scandal and was rejected by the public. Even some of Picasso’s close friends were shocked and outraged. Cognitive mastering was impossible for most of Picasso’s contemporaries, preventing an aesthetic experience. As the public grew more familiar with cubism and modern art, cognitive mastering became possible and art viewers learned to appreciate the intrinsic beauty of this painting. Today, the painting is considered a milestone of 20th century art and enables an aesthetic experience similar to that illustrated in **Figure 4**.

**FIGURE 7:**
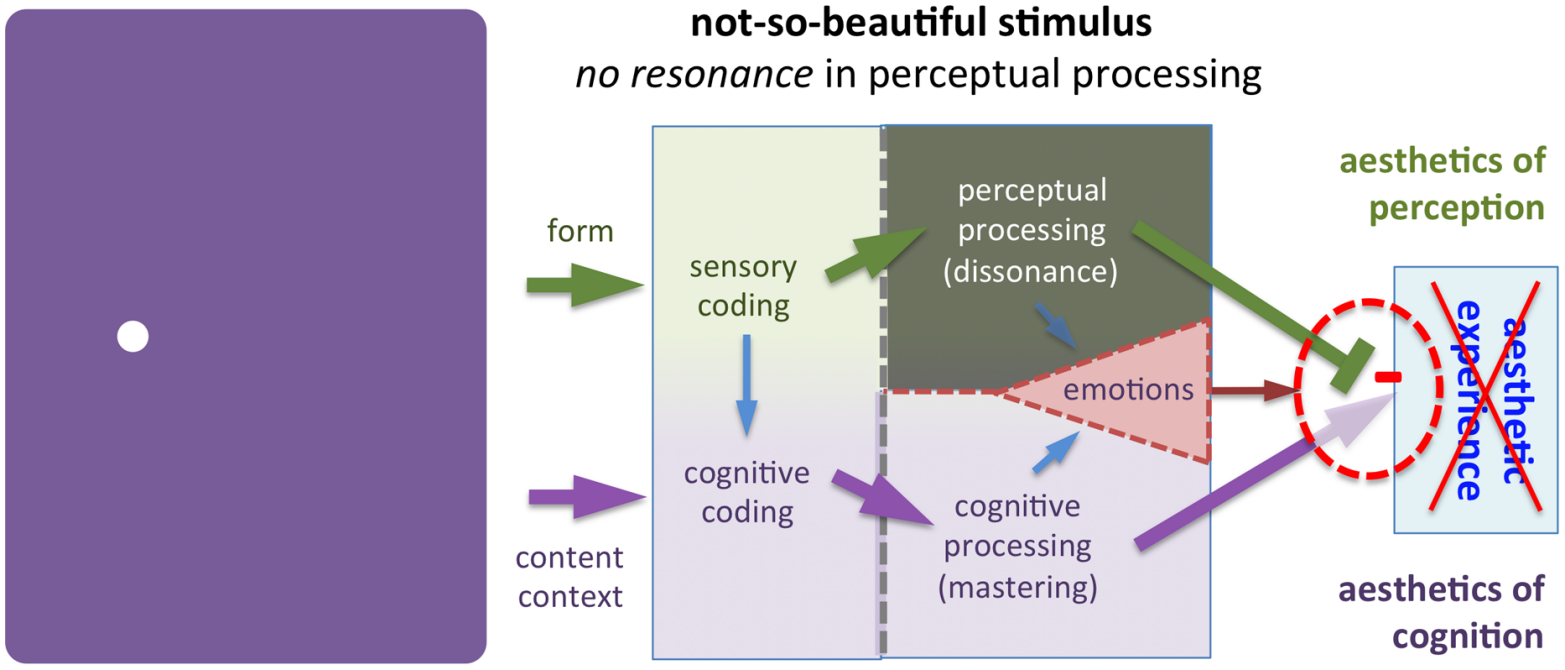
**Example of a not-so-beautiful painting (*Monochrome* by Redies, 2014)**. The author produced this painting on the occasion of the present article. It is *not* a counterfeit of Yves Klein’s (1928–1962) monochrome paintings. Note that the color of the painting is different from Klein’s standard ultramarine blue. Also, it was the artist’s intention to break with the tradition of purely monochrome paintings by superimposing a highly innovative white spot. Even if this explanation might allow some readers to cognitively master the context of this painting, its intrinsic beauty remains negligible. As a result, there will be no aesthetic experience for most individuals. In the model, the gray shading indicates unsuccessful perceptual processing. The occasional reader, who does have an aesthetic experience with this or Yves Klein’s paintings, may fall under the case illustrated in **Figure 10**.

**FIGURE 8:**
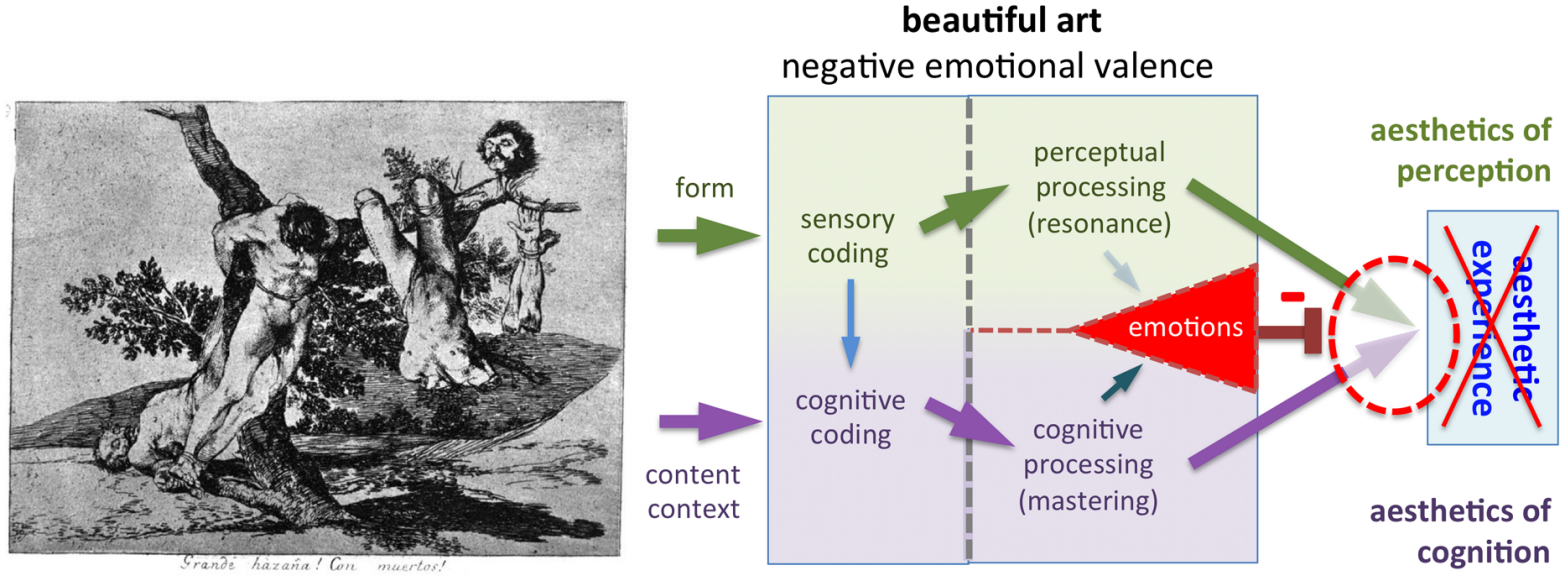
**Example of an artwork with strongly negative emotional valence (*<Grande hazaña! Con muertos!* Plate no. 39 from *Desastres de la Guerra*, 1810 –1814, by F. de Goya)**. Despite the formal beauty of this etching and the pacifist intentions of the artist, this cruel war scene can induce strongly aversive emotions in the beholder, which will block aesthetic experience in most individuals.

**FIGURE 9:**
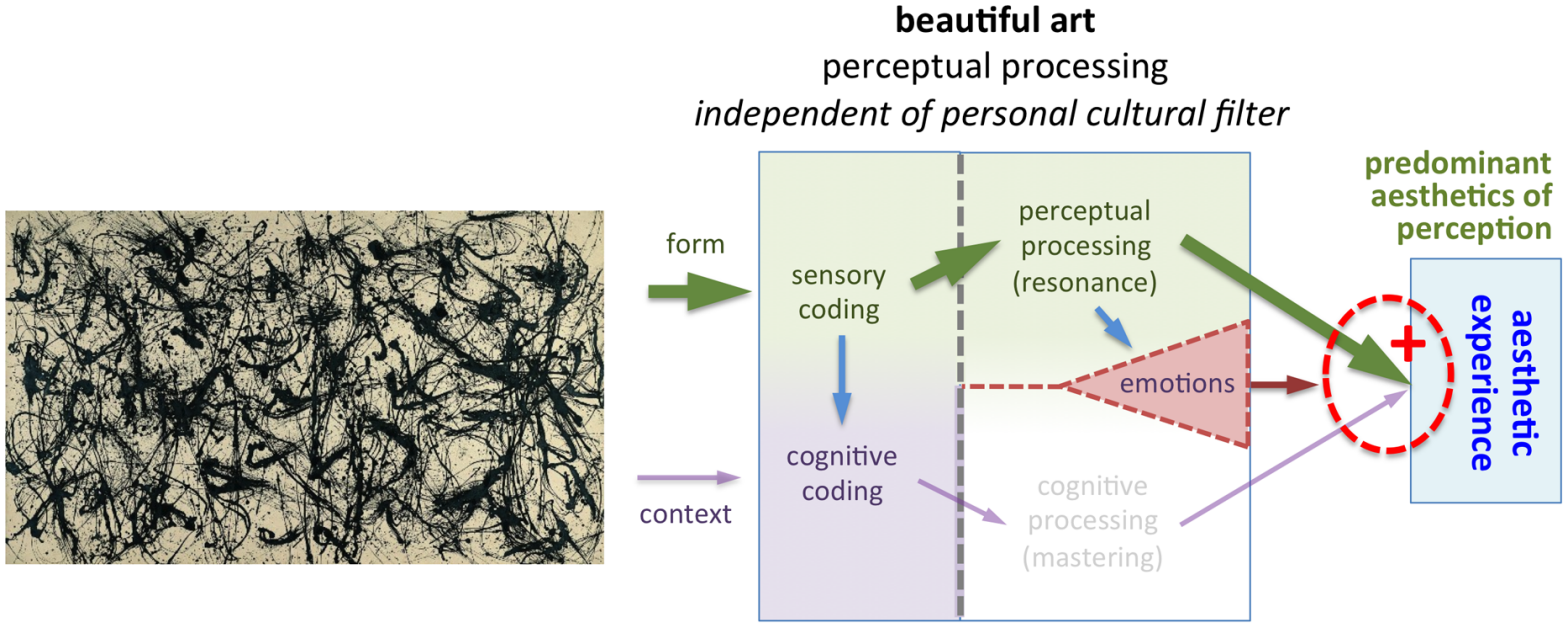
**Example of an abstract expressionist painting (*Number 32* by Pollock, 1950, © Pollock-Krasner Foundation/VG Bild-Kunst and Bonn, 2015)**. Taylor (2002) showed that Pollock created fractal (self-similar) structures in his drip paintings. Although Pollock was aware of the similarities of his paintings and natural forms in general, he did not know about fractals, which were discovered only later by Mandelbrot (1975). The art critic Greenberg (1967) argued that the artistic quality of paintings like those of Pollock can be valued exclusively on the basis of their form. Following this formalist argument, some art beholders can enjoy the beauty of artworks with few or no cognitive reflections about the intentions of the artist and the context of the creation of the paintings. In the model, this is realized by the decrease of cognitive processing (i.e., predominant aesthetics of perception), which is the opposite of the case shown in **Figure 10**.

**FIGURE 10:**
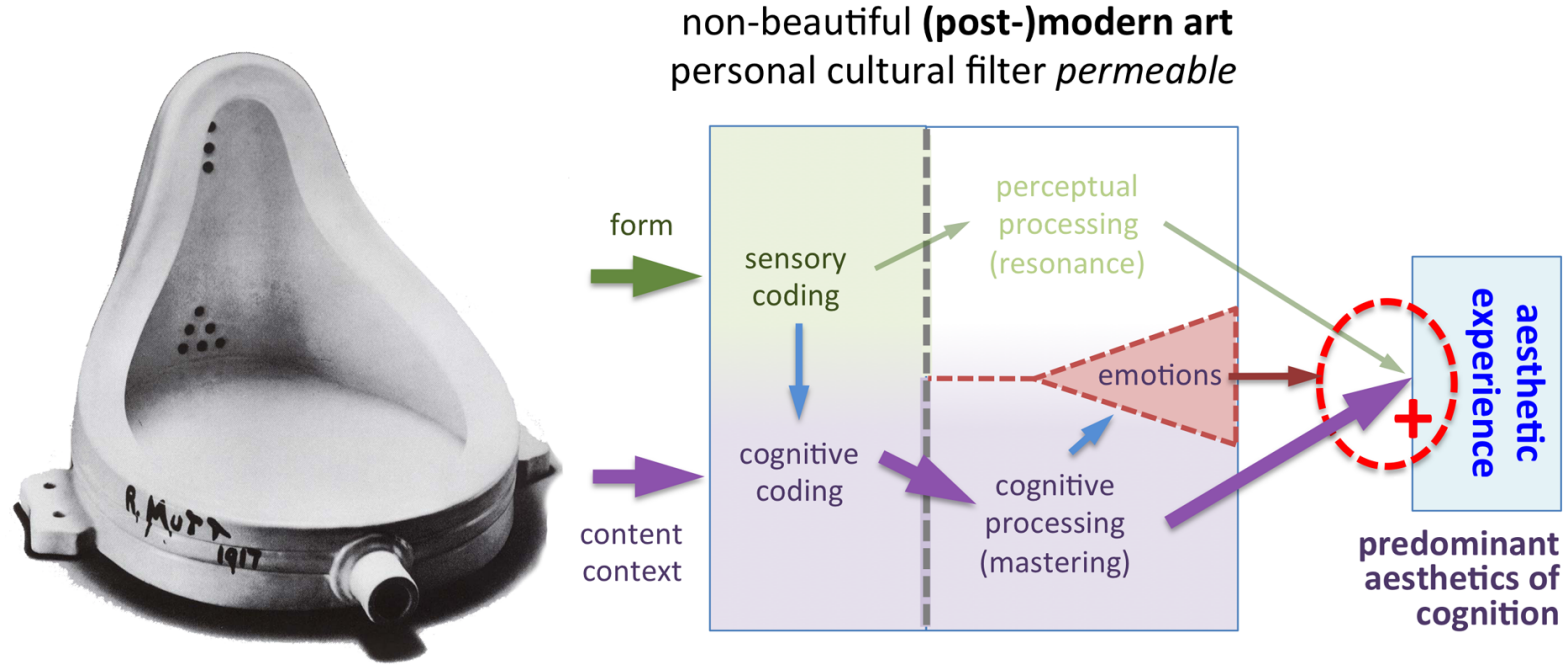
**Example of a modern artwork that lacks visual beauty (*Fountain* by Duchamps, 1917, © Succession Marcel Duchamp/VG Bild-Kunst and Bonn, 2015)**. Many art historians consider this porcelain urinal a major landmark artwork of modern art. Its importance in art history stems not so much from the intrinsic beauty of its form, but from the fact that the artist transcended traditional artistic principles and thereby set the stage for modern conceptual art. In the process of establishing (post-)modern art, many artists abandoned the concept of beauty as a prerequisite for artworks. In the model, this is reflected in the decrease or absence of perceptual processing. As a result, aesthetic experience is determined largely by the content and context of the artwork (i.e., predominant aesthetics of cognition), irrespective of its form.

**Figures 4–10** illustrate how the model works for different types of visual stimuli. A detailed account of each case is provided in the legends to the figures. In labeling the stimuli as beautiful or not, I have tried to subjectively describe the putative judgment of a wide audience. However, some people may disagree with my labels, e.g., they may find the paintings displayed in **Figures 5** and **7** aesthetic.

In brief, if there is resonance in the perceptual channel of the model, as well as mastering and positive evaluation in the cognitive channel, an aesthetic experience is elicited (**Figure 4**). If processing in one or both of the channels is unsuccessful (**Figures 5–7**), there is no aesthetic experience. Strong emotions of negative valence can partially or completely inhibit an aesthetic experience (**Figure 8**).

Note that the examples described are not static but can change over time. For example, individuals can change their cognitive processing due to cultural adaptation. In the case of scandalous but beautiful artworks, the response of art viewers can change from outright rejection (**Figure 6**) to an aesthetic experience (**Figure 4**), as the public grows familiar with novel styles of art. Whether the beauty-responsive mechanism is also subject to temporal modulation is less clear. The perception of beauty in abstract artworks was recently shown to be subject to perceptual contrast, which seems to correlate with specific image properties, such as color measures (Mallon et al., 2014). More detailed studies are necessary to investigate this question.

### Predominant Activation of One Channel

In the following, I will discuss two special cases, where one of the processing streams (perceptual of cognitive) dominates and the other stream plays only a minor or no role in the aesthetic experience.

First, some art viewers may choose to minimize cognitive evaluation while viewing art (*predominant aesthetics of perception*). For example, it has been claimed that viewing Pollock’s painting *Number 32* (**Figure 9**) can be a purely sensual experience that is largely, if not completely, devoid of cognitive evaluation (Greenberg, 1967). For most individuals, however, formal aspects alone may not be sufficient to reach a full aesthetic experience when viewing artworks; they may be compelled also by cognitive factors, e.g., by realizing the revolutionary style of J. Pollock (Rosenberg, 1952) or the high cost of his paintings. Because the perceptual mechanisms of beauty perception do not change much over time, aesthetics of perception is likely to be relatively stable.

What is the evidence that aesthetic experience can be induced predominantly by perceptual processing when, at the same time, cognitive processing is reduced? (i) Persons with dementia show a remarkably stable preference for artworks despite their cognitive decline in the course of the illness (Graham et al., 2013; Gretton and ffytche, 2014). In patients with frontotemporal dementia, artistic production can even be induced or enhanced in some cases (Miller and Miller, 2013; Zaidel, 2014). (ii) Judgments on global and holistic properties of artworks, such as balance, symmetry, structural complexity, and structural arrangement can be reached with presentation times of 50–100 ms, i.e., by gist perception (Cupchik and Berlyne, 1979; Locher and Nagy, 1996; Smith et al., 2006; Locher et al., 2007); this time period is probably too short for extensive cognitive deliberations. Global image statistics that can be correlated with perception of scene gist, such as spatial coherence, are encoded in the visual system within the first 100–150 ms after stimulus onset, indicating processing by early visual areas (lateral geniculate nucleus or primary visual cortex; Kreitler and Kreitler, 1984; Groen et al., 2013). (iii) Some artists claimed that they followed unspecified or subconscious abstract compositional rules while creating their artworks, for example, Kandinsky (1912) and A. Dürer (Panofsky, 1943). Malevich (1927), a pioneer of geometrical abstract art, stated that reality, objects, meaning and context are irrelevant for his artworks. (iv) Babies prefer to look at attractive faces, suggesting that the preference for facial beauty is not driven by cognition but may reflect an inborn mechanism of perception (Valenza et al., 1996; Samuels et al., 2013). However, this effect may be specific for the face domain (see “Higher-Level Perceptual Processing”); it is presently unknown whether there is a similar preference for beautiful artworks. (v) Last but not least, in a psychological experiment, Cupchik and Wroblewski-Raya (1998) differentiated between perceptive *versus* projective functions of art, as initially proposed by Machotka (1979). Perceptive functions pertain to the aesthetic structure of objects (style) while projective functions relate to the observer’s own cognitive and emotional needs. Participants who took a more personally distant view of the paintings preferred the style over the narrative subject matter of the artworks, while persons who found the paintings to be personally meaningful, preferred the subject matter over the style. This result demonstrates that viewers can shift between perceptual and cognitive processing, depending on the context and their own internal needs.

Second, many contemporary art experts argue that the concept of beauty is largely abandoned in modern and post-modern art; instead, the aesthetic impact of artworks is thought to depend on the intention of the artist, the art historical context of the artwork’s creation and the appraisal by art experts (see “Content and Context”). For example, in the model of aesthetic experience by Leder et al. (2004; modified in Leder and Nadal, 2014), an aesthetic episode requires cognitive mastering and positive evaluation of the intentions of the artist and the circumstances, under which an artwork was created and is presented. The expertise of the art beholder supports this evaluative process because it sustains the explicit processing of social, historical and other types of relevant information as well as the beholder’s interest and cognitive stimulation by the artwork. Bullot and Reber (2013) suggested that any explanation and classification of psychological responses to art must be based on the study of art-historical information, besides psychological factors. The art critic Gopnik (2012) goes even further and contends that the role of the art critic is to instruct the general public on how to understand and appreciate artworks.

The above notion has been criticized as parochial and elitist (Redies, 2014). It seems parochial because not all modern, post-modern or contemporary artworks lack visual beauty. On the contrary, some (post-)modern artworks are highly beautiful in the sense that their visual sensual qualities tend to dominate the aesthetic experience by most individuals (aesthetics of perception; **Figure 1**). Examples are artworks by V. Kandinsky, P. Picasso, J. Miro, J. Pollock, and others. Moreover, artists like M. Rothko, B. Newman, and A. Gottlieb have advanced formalist concepts in their art (Greenberg, 1955; Dowling, 2014). According to them, the inherent visual quality of their artworks determines their aesthetic value. Moreover, it is elitist to claim that experts enjoy artworks at a generally higher level than non-experts because of their superior factual knowledge or cognitive abilities (Fitch and Westphal-Fitch, 2013).

Despite these caveats, there is no doubt that many 20th/21th century artworks are less reliant on visual beauty, and that they can nevertheless evoke an aesthetic experience in some experts, based on cognitive mastering and evaluation. In aesthetics of cognition, “the challenge of art is mainly driven by a need for understanding” (Leder et al., 2004). One of the prototypical examples of this type of art is M. Duchamp’s *Fountain* (**Figure 10**). Note that visual stimuli that are considered artworks at a given time can loose their art status if the favorable conditions for cognitive mastering are no longer given at another point in art history or at another place in the cultural environment. As a consequence, aesthetics of cognition comes along with some degree of momentariness.

It may seem contradictory that, on the one hand, the model (**Figure 1**) requires activation of both channels for an aesthetic experience in the general case, and that, on the other hand, the predominant activation of one channel seems to suffice in the extreme cases of aesthetic experiences described above. While this may be viewed as a conceptual weakness of the model, note that the extreme cases do not require additional modeling but derive from parts of the model. The model is thus flexible with regard to individual differences, which are so conspicuous in art perception.

### Emotional Processing

Emotional processing is generally considered to be another important component of aesthetic perception (Jacobsen, 2004; Leder et al., 2004; Chatterjee and Vartanian, 2014; Leder and Nadal, 2014; Silva, 2014). Emotions can play a role both during the creation of artworks and their viewing. Artists communicate their emotions through the artworks to the beholder. Artworks can induce basic (primary) emotions, such as happiness, melancholy, disgust, fear, anxiety, both by their perceptual properties and by their content, and they do so in a particularly effective manner. However, there is general agreement in the literature that aesthetic experience is not one of the primary emotions. A well-known version of this notion is the concept of “disinterested pleasure” by Kant (1790). It is still debated and perhaps a matter of definition whether the aesthetic experience can be regarded as a specific emotion or affectual state. For example, Leder et al. (2004) distinguish aesthetic emotion and aesthetic judgment as two separate outcomes of aesthetic processing. Other authors even consider emotions as central to artistic activity (Silva, 2014) or proposed that the role of artworks is to express emotions (for a review, see Shimamura, 2014).

In my model, primary emotions that are expressed in artworks can have a modulatory effect on the aesthetic experience of the beholder. Both the perceptual processing and the contextual processing may induce emotions (**Figure 1**). For example, the perception of bright and saturated blue and yellow colors in artworks may enhance aesthetic experience, while brownish colors may induce aversion in art beholders and thus cause a decrease of aesthetic experience in general (Yanulevskaya et al., 2012; Palmer et al., 2013; Mallon et al., 2014). Moreover, as another example, people generally prefer everyday objects with curved contours to objects with sharp-angled contours or pointed features (Bar and Neta, 2007). Emotions induced by image content may also modulate aesthetic experience. For example, most people show a fearful emotion in response to images of spiders and snakes and this fear represents an inborn mechanism (for a review, see Öhman, 2008). In extreme cases, particularly gruesome or disgusting visual scenes induce feelings of repulsion and disgust that can block aesthetic experience altogether, for example, scenes of war (**Figure 8**).

However, emotional processing of positive valence is not a prerequisite for aesthetic experience and the extent, to which emotions can affect the aesthetic preference for artworks, varies between individuals (Konecni and Sargent-Pollock, 1977). Likewise, negative valence of emotions does not necessarily mean that aesthetic experience is abolished in everyone. In particular, some music listeners enjoy music even if it evokes sadness; in these listeners, the liking of sad music has been associated with particular character traits (Vuoskoski et al., 2012). In conclusion, the experience of emotions may modulate aesthetic experience but it is not a necessary or sufficient condition to induce it.

### Aesthetic Experience

The final stage of the model is the aesthetic experience, which takes place if the two processing modalities (perceptual and cognitive) meet the specific provisions outlined above (see “Joint Activation of Perceptual and Cognitive Channels” and “Predominant Activation of One Channel”).

Recent brain imaging studies demonstrated that aesthetic experience in different sensory or cognitive domains, for example, in visual art, music, gustation/olfaction, touch and even mathematics, leads to the activation of a similar set of brain regions. These regions include the orbitofrontal cortex, a brain region associated with the reward system and moral judgments (for reviews, see Brown and Dissanayake, 2009; Avram et al., 2013; Ishizu and Zeki, 2013; Chatterjee and Vartanian, 2014; Vartanian and Skov, 2014; Zeki et al., 2014). Another region, the anterior insula, was identified in a meta-analysis of 93 neuroimaging studies across four different modalities (Brown et al., 2011). Because both regions receive visceral afferents, the authors speculated that aesthetic appreciation was originally associated with objects such as food and was later co-opted for the experience of artworks in social contexts. Last but not least, some of the brain regions that are activated by observing artworks are associated with the default mode network, a set of regions that are active during rest and self-referential thought; this finding may correlate to the feeling of being touched “from within” by artworks (Vessel et al., 2013).

Although the activation of the above-mentioned regions seems to correlate with aesthetic experience across domains, their functional mapping may contribute little to our understanding of what is special about beautiful stimuli and how these special characteristics are processed in the brain. By analogy, studying the motor brain areas that control the muscles active when a person is laughing would add little to our understanding of what is funny about jokes. In this sense, it can be questioned whether the universal essence of aesthetic experience lies in the activation of these regions, as proposed by Zeki (2013). Rather, these brain regions may represent a common endpoint where more widespread mechanisms of aesthetics converge from different sensory and cognitive systems in the brain.

## Implications of the Model and General Discussion

### Potential Role of Beautiful Art in Social Bonding

To understand the relevance of the parallel (perceptual and cognitive) processing streams, it may be helpful to reflect upon the role of art in pre-modern, aboriginal societies, the social structure of which resembles that of our human ancestors who lived at the time when art practice originated in the human lineage about 30,000 years ago (Valladas et al., 2001). Descriptions of art practice in such small-scale societies suggest that art promotes social bonding between the members of a group of pre-modern people (Eibl-Eibesfeldt, 1988). In a similar vein, Dissanayake (2008) proposed that, in human evolution, art served the adaptive function of reinforcing sociality, enhancing cooperation and contributing to social cohesion and continuity. Brown and Dissanayake (2009) argued that this bonding function of art practice, especially when carried out in conjunction with ritual ceremonies, is probably more basic than other functions proposed in the field of evolutionary aesthetics (Dissanayake, 2007), for example the promotion of mating opportunity through the display of honest signals of reproductive fitness (Voland, 2003) or the aesthetic preference of early humans for certain natural habitats (savannah hypothesis, Orians, 1986). In this context, it is of interest that the neural correlates of aesthetic experience partially coincide with a neural network in the brain that is also involved in moral judgments (Zaidel and Nadal, 2011; Avram et al., 2013); this linkage may be the basis of the beauty-is-good stereotype (Tsukiura and Cabeza, 2011).

**FIGURE 11:**
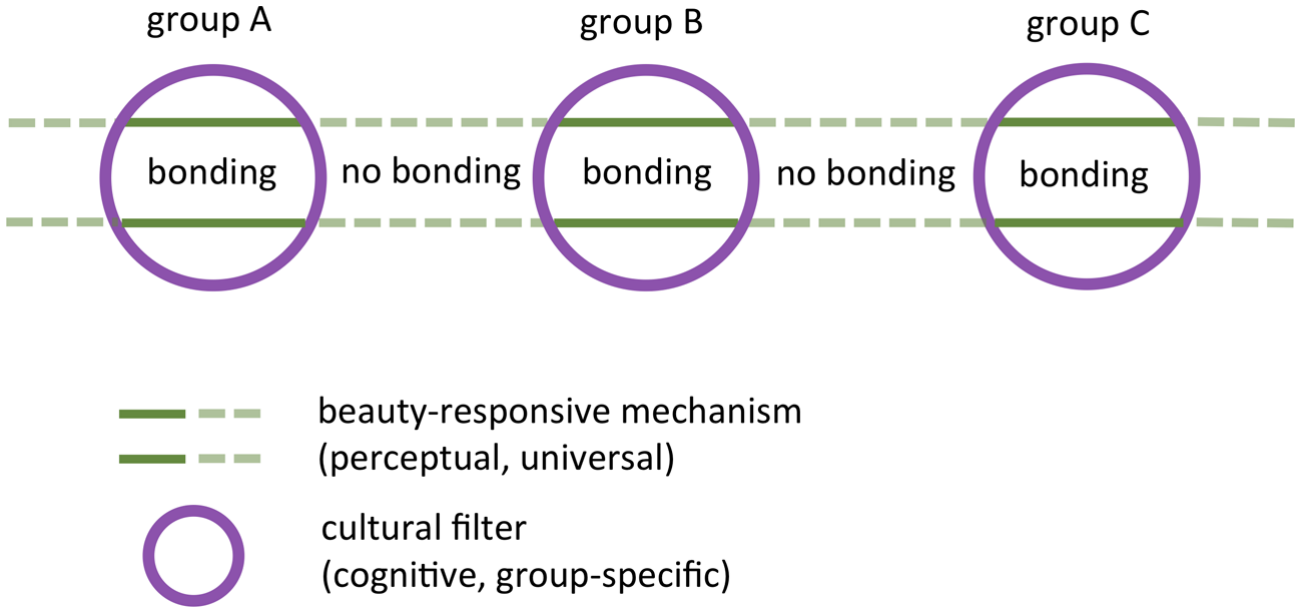
The role of beautiful artworks in group-specific social bonding. Bonding is mediated by a combination of two concurrent mechanisms. It is induced by the activation of a universal perceptual mechanism by beautiful visual stimuli (*beauty-responsive mechanism*; green lines), but only if the beholder is tuned to the cognitive attributes of the stimulus (*cultural filter*; purple circles). In this way, social bonding takes place between members sharing a specific culture, but not between members of different cultural groups.

In the model, social bonding is based on the perceptual processing of beautiful form (see “Perceptual Coding and Processing”), which is postulated to be universal among all cultures (green lines in **Figure 11**). Social bonding comes about if the members of a group enjoy beautiful visual displays such as artworks together, for example in ceremonies. Beauty may thus serve as a cultural “glue” for social bonding. Being universally recognized, however, it is unspecific with respect to cultural identity. In general, social bonding takes place between persons who belong to the same group but not between members of different cultural groups. The mechanism responsible for social segregation and cultural identity is the personal cultural filter (purple circles in **Figure 11**). The filter is permissive if and only if the beholder is familiar with the artwork’s content and can understand the context of its creation and presentation. This is likely to be the case if he is a member of the same group (for example, group B in **Figure 11**). If he is a member of another group (group A or group C), he will be ignorant of some of the cultural details of group B. As a result, cognitive evaluation of the artwork is not possible and the cultural filter remains blocked so that aesthetic experience fails and there is no social bonding. Therefore, the role of cognitive processing in the model is to promote the bonding between members of the same group, but to inhibit the bonding between members of different social groups. A modulatory effect of primary emotions on aesthetic perception (see “Emotional Processing”) seems plausible because social bonding constitutes a positive reinforcement of social interactions that would be adversely affected by negative emotions in general. In modern (large-scale) societies, which each produce their characteristic cultural artifacts, including artworks, these mechanisms may still be in place.

### Does the Beauty-Responsive Mechanism Differ between Individuals?

Because cognitive processing depends on the individual exposure to the cultural environment, the personal cultural filter varies to a great extent between individuals. It is less clear, however, whether individuals also differ in the extent, to which the beauty-responsive mechanism reacts to beautiful artworks. In Section “What is the Nature of the Beauty-Responsive Mechanism and Which Stimulus Features Activate It?” I argued that the beauty-responsive mechanism relates to basic ways of neural processing and is universal amongst humans. Can such a mechanism possibly differ quantitatively between individuals? Results for other domains of social recognition suggest that the answer to this question might be affirmative. For example, face recognition is a universal psychological mechanism among humans. The neural correlates of face recognition have been studied extensively. Like for the perception of beauty, there is evidence that both inborn universal mechanisms and culturally determined factors (e.g., the other-race effect) play a role. It is also clear that human differ in their ability to recognize and memorize faces, ranging from deficits (called prosopagnosia) to super-recognizers, who perform considerably better than average in face recognition (Russell et al., 2009; Wilmer et al., 2012). Other traits of social recognition, for example, autistic traits, are thought to vary widely in the general population also (Constantino and Todd, 2003; Robinson et al., 2011). Both conditions are subject to genetic predisposition. It is therefore possible also that individuals differ in their ability to perceive visual beauty. Indeed, such differences are suggested by the widely varying views of people on how important beauty is for aesthetic perception, ranging from negligible to essential. However, to date, little is known about the extent of such individual differences. In order to study them, reliable tests with suitable stimuli must be developed first (for example, see Wilson and Chatterjee, 2005; Wilmer et al., 2012).

A related question is whether, in general, artists and art viewers differ in their ability to perceive beauty. I speculate that this does not necessarily have to be the case. Usually, artists create artworks under a constant feedback between the emerging artwork and their own visual system. The unique skill of artists might lie in their ability to create images that induce a beauty-related, resonant state in the visual system of human beholders. To create beautiful art, artists must have the technical skills, visual sensitivity and mental imagination to predict which steps in the production of an artwork bring them closer to a maximal activation of the beauty-responsive mechanism. The difference between an artist and art beholders may thus lie in the artist’s creativity, imagination and skills rather than in the extent, to which he or she perceives beauty in artworks.

### Comparison with Other Models

The present model follows previous suggestions that aesthetic experience is based on the triad of perception, cognition and emotion (Leder et al., 2004; Chatterjee and Vartanian, 2014). In a previously proposed multi-stage model, which was developed primarily to explain modern and contemporary art (Leder et al., 2004; Leder and Nadal, 2014), perceptual processing begins at low levels of the model. It is followed by cognitive processing of style, content and context at higher levels, eventually leading to cognitive mastering, understanding and evaluation, which are based mainly on acquired expertise. In contrast to such hierarchical processing (see also Locher et al., 2007), the present model assumes parallel and independent processing of perceptual aspects of artworks, which are universal among humans, and cognitive aspects, which depend on individual experience and cultural context. The two processing streams converge at the level of aesthetic experience. The seeming conflict between perceptual and cognitive processing is resolved by assuming that both types of processing must be successful in order to elicit an aesthetic experience in the general case (see “Joint Activation of Perceptual and Cognitive Channels”). Previously, Sheridan and Gardner (2012) argued that psychological studies of the arts should not be restricted to bio-psychological universals but also take into account culture and individual differences as a modulatory influence to account for the full richness of artistic production and aesthetic preferences. Other authors have been less specific about how to integrate perceptual universals and contextual dependencies of art perception (Jacobsen, 2006; Chatterjee and Vartanian, 2014).

The role of emotions in the present model is a modulatory one. In the Leder et al. (2004) model, affective evaluation occurs continuously and in parallel to the processing of explicit information, leading to an aesthetic emotion in addition to aesthetic judgment as outputs from their model. A close interplay between perceptual processes and emotions is also pivotal to the predictive coding model of visual art proposed by Van de Cruys and Wagemans (2011). In their model, artists destroy initial predictions of the art viewers who then feel rewarded after recovering predictable patterns again on a different level; reward is thus derived from the transition from a state of uncertainty to a state of increased predictability.

Compared to the previous models, the present model does not necessarily lead to more numerous or more specific predictions. However, the model leads to a set of specific predictions that is different from previous models, especially with regard to the role of beauty and its relation to cognition in aesthetic experience. For example, the present model makes predictions on the possible nature of the putative beauty-responsive mechanism and its localization in the brain, inter-individual differences in aesthetic experience, the difference between the beautiful form of visual stimuli and more domain-specific visual preferences, the modulatory role of emotions, and the evolutionary origin of aesthetic experience and animal aesthetics (see above and below). In a young field of research like experimental aesthetics, where a sizeable body of data is still missing, it is good to have a diversity of models rather than a single one because it is important to keep an open mind for all types of possible research questions (see Introduction). The results from future experiments will tell us which of the different models has the highest predictive power.

### Application of the Model to Other Areas and Domains of Aesthetics

The present model was designed to describe and explain aesthetic experiences in response to visual artworks. Can the model be applied to a wider array of aesthetic phenomena? To answer this question, I will consider four types of extensions of the model.

First, for most people, the aesthetic evaluation of artworks is a rare event in everyday life. More frequently, an individual encounters other types of complex visual stimuli that may be beautiful to varying degrees, such as clothing, advertisements, architecture, design etc. The model can be applied also to these types of visual stimuli. In particular, the interacting roles of beauty and contextual (cultural) factors in promoting cultural identity and bonding (**Figure 11**) is evident for aesthetic experiences that are subject to fashion trends, such as for clothing and design. Interestingly, the statistical image properties of artworks were shown to overlap to a large degree with those of advertisements and images of architecture in Western culture (Braun et al., 2013). These types of images and other naturally occurring images are relatively complex and possess a nearly scale-invariant Fourier spectrum (i.e., power-law behavior of their spatial frequency spectrum) in general (for a review, see Simoncelli and Olshausen, 2001).

Second, more simple, man-made visual stimuli rarely occur in our natural environment in isolation. They include geometrical forms (McManus, 1980), simple graphic patterns (Jacobsen and Höfel, 2002) or large fields of homogeneous color (Palmer and Schloss, 2010). These stimuli generally possess other types of image statistics, as demonstrated for images of regular text (Melmer et al., 2013). The human visual system is adapted to process images with natural scene statistics in a particularly efficient way (see “Form”), but it may respond in a different way to images with non-natural image statistics. For example, it has been shown that artificial images that can induce visual discomfort in human observers deviate from the scale-invariant Fourier spectrum of natural scenes (Juricevic et al., 2010; O’Hare and Hibbard, 2011). It is therefore possible that the perception of beauty in naturally looking stimuli, such as beautiful artworks, differs from the visual preference for other types of stimuli, such as simple, highly geometrical patterns. In how far the present model can be applied to such stimuli remains to be studied.

Third, some visual stimuli are beautiful in a more special (domain-specific) sense, such as images of human faces, human bodies or landscapes. **Figure 3** illustrates the difference between the beauty (attractiveness) of faces and the beauty (global composition) of artworks. On the one hand, it is well established that facial attractiveness is based both on universal (inborn) perceptual mechanisms and cultural influences. In this respect, the perceptual mechanisms of facial attractiveness and beauty in artworks follow a similar ontogenetic pattern. On the other hand, it is also clear that facial attractiveness is dependent on a particular class of objects (i.e., human faces) while the beauty of artworks does not depend on the content depicted in the artworks (see “What is the Nature of the Beauty-Responsive Mechanism and Which Stimulus Features Activate It?”). Evidently, face attractiveness is relevant for mate selection, as is the beauty of the human body. It is unclear why the image statistics of natural scenes may have generalized to be associated with content-independent beauty while those of faces have not. The preference of artists for specific image properties (Redies et al., 2007a) shows that beauty in artworks differs from content-dependent (facial) beauty not only behaviorally but also in terms of physical stimulus properties. Nevertheless, the beauty of landscapes and the beauty of artworks are not identical. By creating artworks, artists can implement other, as of yet unknown regularities beyond natural scenes statistics, perhaps to induce a deeper or more complete sense of beauty. In this way, artworks may reflect additional mechanisms of neural processing intrinsic to the visual system, such as efficient coding (Redies, 2007).

Fourth, although the model was introduced for the visual domain, it can be extended to aesthetic experiences in other sensory domains. These domains differ substantially along several parameters of their processing architecture, but they may also share commonalities (Jacobsen, 2014). For music perception, which contains relatively little semantic content, aesthetics of perception and emotional arousal are particularly relevant. In music, some universals have already been identified, such as timekeeping during music performance (for a review, see Palmer, 1997), pitch perception and harmonic relations (for a review, see Koelsch, 2011). Egermann et al. (2015) found universal arousal responses to low-level acoustic characteristic of music in a cross-cultural study of Pygmies and Canadians. However, aesthetics of cognition may also play a role in music perception because, for example, music style depends on the cultural environment and familiarity with style is an important factor in the appreciation of music. In literature, aesthetics of cognition is of paramount importance. Aesthetics of perception may express itself in prosody, intonation, and rhyme. In Section “What is the Nature of the Beauty-Responsive Mechanism and Which Stimulus Features Activate It?” I speculated that the perception of beauty is mediated by basic mechanisms of neural processing that are widespread in the visual system. Following this notion, it is possible that the same or similar basic mechanisms are implemented also in the other sensory systems in the brain. If this were true, one would expect similar types of beauty-related stimulus structure, for example self-similar (fractal) patterns, across sensory modalities. Indeed, fractal structure has been demonstrated for particular types of music (Voss and Clarke, 1978; Hsu and Hsu, 1990; Su and Wu, 2007; Brothers, 2009), but these findings remain anecdotal and await a more systematic investigation across different types of music and cultures. In the area of brain imaging, comparative studies of different domains are more advanced. For example, the activation of the medial orbitofrontal cortex correlates with the perception of beauty in art, music and even mathematical equations (Zeki et al., 2014), as well as the goodness of moral judgments (Tsukiura and Cabeza, 2011). Last but not least, one may ask whether the cognitive channel contains analogs of the beauty-responsive perceptual mechanisms. If there is beauty of cognition, does it have similar neural correlates in the brain as the perception of visual beauty?

## Other Implications of the Model

Provided that the model correctly represents the factors and mechanisms that play a role in visual aesthetic experience, it has the following additional implications:

(1) According to the model, the beauty-responsive mechanism is implemented in several visual channels or brain regions, which process different aspects of visual information. Unlike the final aesthetic experience, which seems to activate a set of restricted brain regions and neural networks (see “Aesthetic Experience”), the neurophysiological correlate of beauty perception may not be restricted to a single channel or a limited number of brain regions. Moreover, if the beauty-responsive mechanism reflects basic principles of neural processing, such as efficient coding (Redies, 2007), the activation (or inhibition) of the involved neural circuits may take place at a resolution below that of conventional methods to measure brain activity, such as fMRT, MEG, or EEG. It is thus possible that such neural mechanisms escape conventional brain imaging or neurophysiological methods.
(2) If perceptual processing of beauty and cognitive processing of context and content take place along independent channels and in parallel, the beauty-responsive mechanism can be activated even if cognitive processing fails and there is no aesthetic experience. Also, the beauty-responsive mechanisms should be detectable independent of basic emotions, which modulate aesthetic experience at a later processing stage in the model (**Figure 1**).
(3) Can animals have an aesthetic experience? It seems reasonable to assume that cognitive processing of cultural information is not as complex in animals as in humans in general (Tomasello, 1999), but cultural behavior has been observed at least in some species, for example, for whale songs (Mercado et al., 2005; Cantor and Whitehead, 2013). Moreover, pigeons can be trained to discriminate between art styles and between children’s paintings that were classified as beautiful or ugly, respectively, by adult human observers (Watanabe, 2011). If perceptual processing of beauty is basic and universal amongst humans, there is no good reason why this concept should not be extended to animals (Welsch, 2004). To prove that animals can appreciate beauty will be difficult because we cannot communicate with them about beauty or aesthetics. Nevertheless, we can search for neural mechanisms in animals that correspond to the neural mechanisms associated with aesthetic experience in humans. The demonstration of beauty-related brain functions in animals would be of great interest because it would give us insight into the evolutionary origins of aesthetic experience. Moreover, if humans and animals share beauty-responsive mechanisms to some extent, this may explain why there can be cross-species appreciation of beautiful patterns, e.g., the appreciation of elaborate bird plumage or bird songs by humans.

## Conflict of Interest Statement

The author declares that the research was conducted in the absence of any commercial or financial relationships that could be construed as a potential conflict of interest
